# Renal angiomyolipoma with IVC thrombus: A case report

**DOI:** 10.1016/j.ijscr.2020.04.076

**Published:** 2020-05-08

**Authors:** Pietro Kheir, Maher Abdessater, Joey El Khoury, Rody Akiel, Charbel El Hachem, Nabil Tawil, Rahgid El Khoury

**Affiliations:** a Department of Cardiovascular Surgery, Notre Dame des Secours University Hospital Center, Byblos City, Lebanon; b Departent of Urology, Notre Dame des Secours University Hospital Center, Byblos City, Lebanon; c Faculty of Medicine and Medical Sciences, Holy Spirit University of Kaslik (USEK), Jounieh, Lebanon

**Keywords:** Angiomyolipoma, Inferior vena cava, Thrombus, Case report

## Abstract

**Introduction:**

Renal angiomyolipomas (AML) are often regarded as benign tumors. This article reports the case of a 47 year old patient with AML associated with a venous invasion of the inferior vena cava.

**Presentation of case:**

Our 47 year old male patient presented for flank pain and hematuria. CT Scan revealed a 3 cm lobulated low-density lesion in the renal sinus, middle and upper lobes of the right kidney, with evidence of IVC thrombus on angioscan. Right radical nephrectomy and thrombectomy were successfully done with use of cardiopulmonary bypass. On pathology, AML was confirmed.

**Discussion:**

Renal AML are unilateral and sporadic in most cases, with a female predominance. The occurrence of venous invasion in patients with AML is a rare and unique phenomenon. CT scan is the imaging of choice in such cases and surgery remains the optimal treatment.

**Conclusion:**

More focus should be put on the ability of AML to invade venous structures. Early imaging and therapeutic planning are necessary for the best outcome in case of vena cava invasion. Collaboration of the urologist with the vascular surgeon can lead to better surgical results.

## Highlights

• The occurrence of venous invasion in patients with renal angiomyolipoma is a rare and unique phenomenon.
• CT scan is the imaging of choice in case of invasion of vena cava.
• Collaboration of the urologist and the vascular surgeon is sometimes mandatory for optimal surgical treatment.

## 1 Introduction

Though venous invasion is a common complication of renal cell carcinoma (RCC), it rarely occurs in renal angiomyolipoma (AML) given its benign nature. AML consists of three different components in a mixed fashion: fatty tissue, dystrophic blood vessels and smooth muscle: All three components are present in 76% of patients with AML [1]. Venous invasion of the inferior vena cava (IVC) in renal AML is a rare phenomenon, and only 90 case of venous involvement, 76 of which are an invasion of the IVC, have been reported. In this article, which has been reported in line with the SCARE criteria [2], we present a case of renal AML with an IVC invasion in a 47 year old patient.

## 2 Case report

Our 47-year-old man previously diagnosed with right renal AML presented for intermittent right flank pain and gross hematuria. He had no signs or symptoms of tuberous sclerosis complex (TSC). His only relevant past medical history was old peritonitis following an appendectomy.

**Fig. 1:**
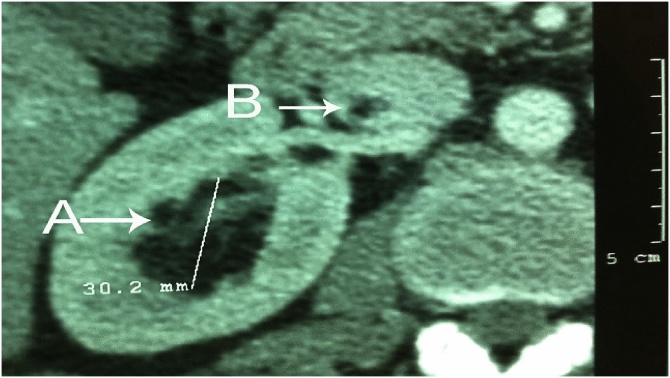
Axial section of abdominal CAT scan showing. A. Multilobulated hypodense (fatty tissue density) in the right kidney. B. Hypodense signal in the IVC, sharing the same fatty density as the tumor.

**Fig. 2:**
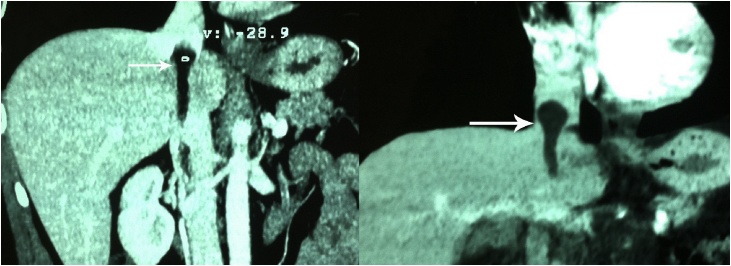
Coronal sections of abdominal angioscan revealing low density lesion extending into the IVC, reaching above the diaphragm superiorly.

The abdominal US showed a hyperechogenic lesion in the right renal sinus with a thrombus in the right renal vein and IVC. Abdominal computed axial tomography (CAT) scan revealed a 3 cm lobulated low-density lesion in the renal sinus, middle and upper lobes of the right kidney (Fig. 1). On abdominal angioscan, the low-density lesion extended from the right kidney into the right renal vein and IVC, reaching above the diaphragm superiorly, 3 cm below the right atrium (Fig. 2).

Considering the risk of pulmonary embolism due to IVC thrombus, the decision of right radical nephrectomy and thrombectomy was taken with the patient.

**Fig. 3:**
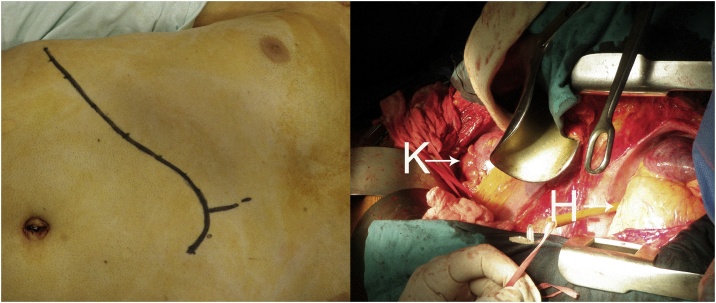
Using a median sternotomy combined with a right subcostal incision, extended shortly towards the left, we had good exposition of the right kidney (K) and the heart (H) and control over the inferior vena cava.

**Fig. 4:**
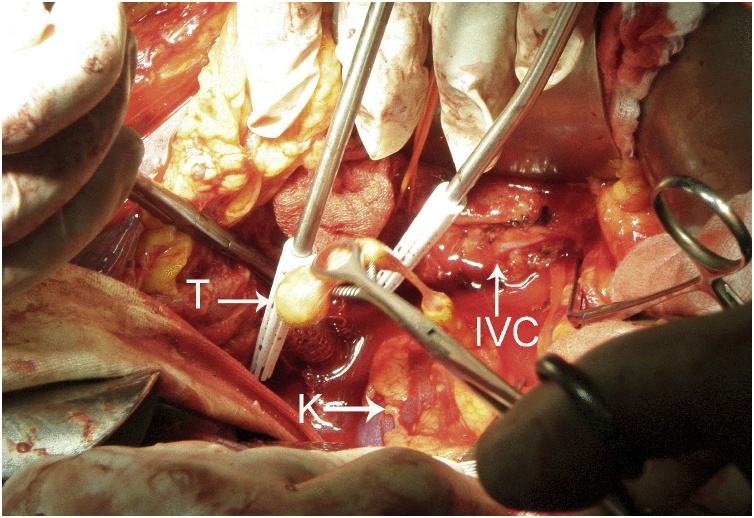
After cavotomy of the inferior vena cava (IVC), the tumor (T) is taken out in one piece with the right kidney (K).

**Fig. 5:**
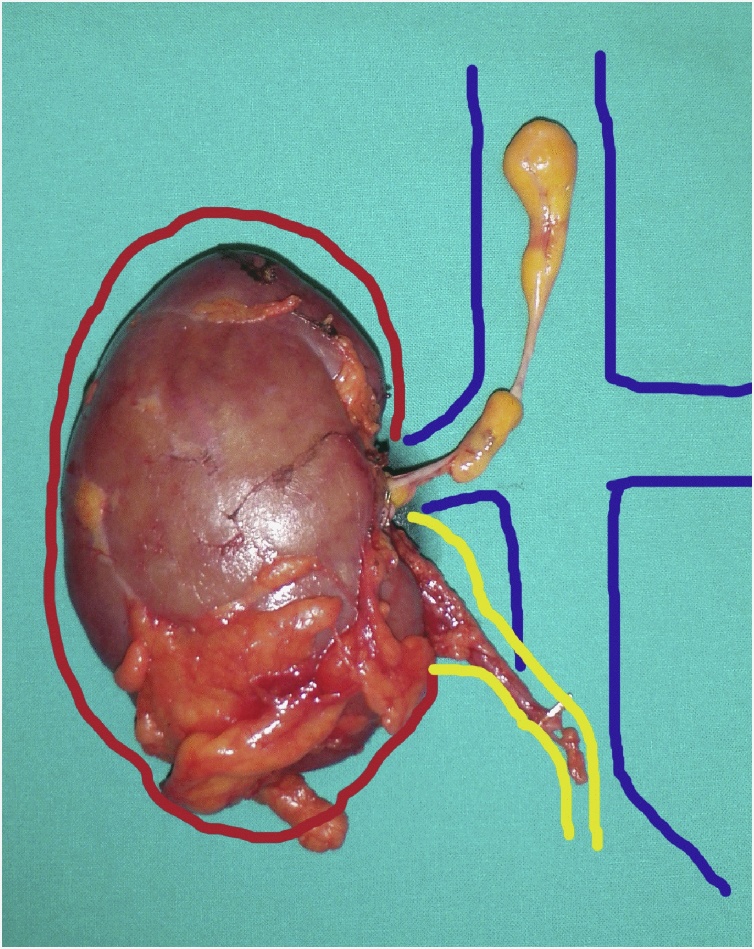
Nephrectomy-thrombectomy piece showing the kidney (red), renal pelvis and ureter (yellow) and tumor thrombus extending through the right renal vein into the inferior vena cava (blue), in anatomical positions.

**Fig. 6:**
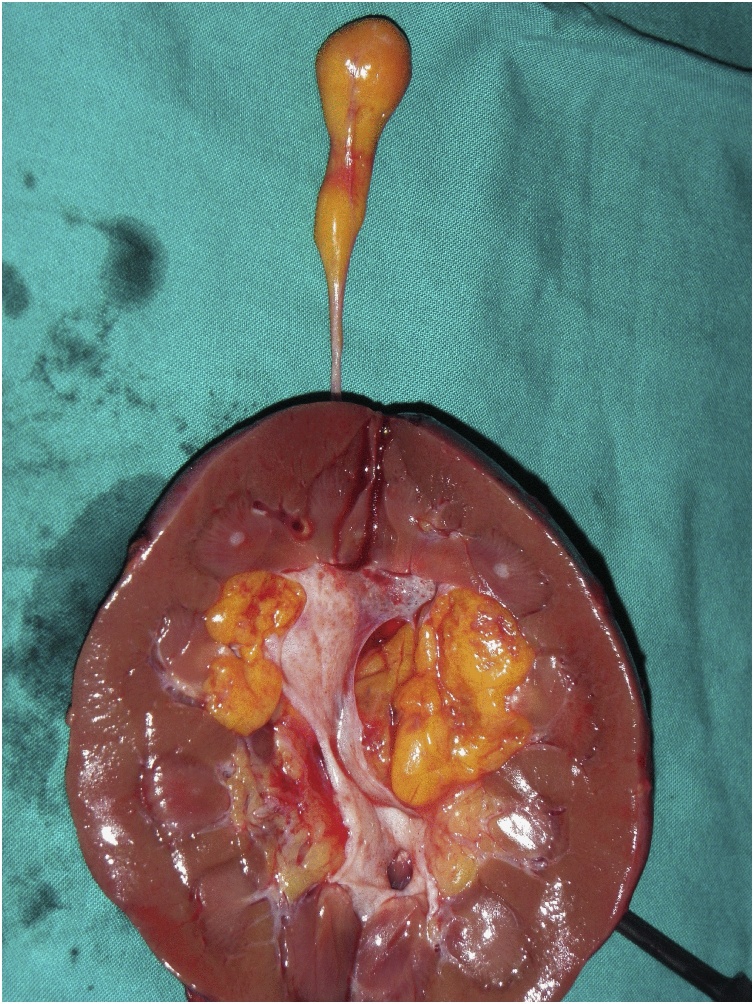
Coronal incision of the right kidney revealing the fatty tumor in the renal pelvis. The thrombus here is shown behind the kidney and is of the same gross appearance as the renal tumor.

**Fig. 7:**
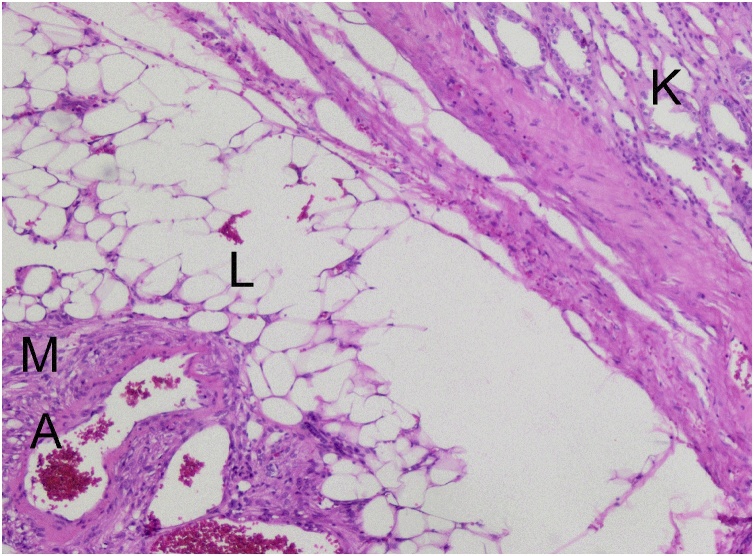
Pathology section showing normal kidney parenchyma (K), and clear delimitation from the tumor consisting of blood vessels (A), smooth muscle cells (M) and fat (L).

The surgery was done under general anesthesia after central and arterial lines insertion, the patient was in dorsal decubitus position. Right subcostal approach with median sternotomy was used (Fig. 3). After isolation of the right kidney by dissection and ligation of the ureter, renal lobar arteries, and collateral veins, leaving intact and minimally mobilizing the right renal vein, cardiopulmonary bypass (CPB) was initiated. A single-stage venous cannula was inserted through the right atrium into the superior vena cava, and ascending aortic cannulation was used for arterial return. Using Satinsky clamps the vena cava was clamped right beneath the right atrium and beneath the renal veins. A 5 cm cavotomy revealed a free-floating pedunculated tumor extending from the right renal vein into the IVC, around 10 cm upwards towards the supradiaphragmatic IVC. The tumor was taken in one piece with the right nephrectomy (Fig. 4, Fig. 5, Fig. 6, Fig. 7). After repair of the cavotomy with running 4–0 Prolene sutures, CPB was interrupted after 28 min of initiation. The right adrenal gland was spared. A drain was put in the peritoneum at the end of the surgery, the operative time was 200 min, the blood loss was 550 cc and no transfusion was needed. The drain was removed at the second post-operative day and the patient was discharged uneventfully on the seventh day after surgery

**Fig. 8:**
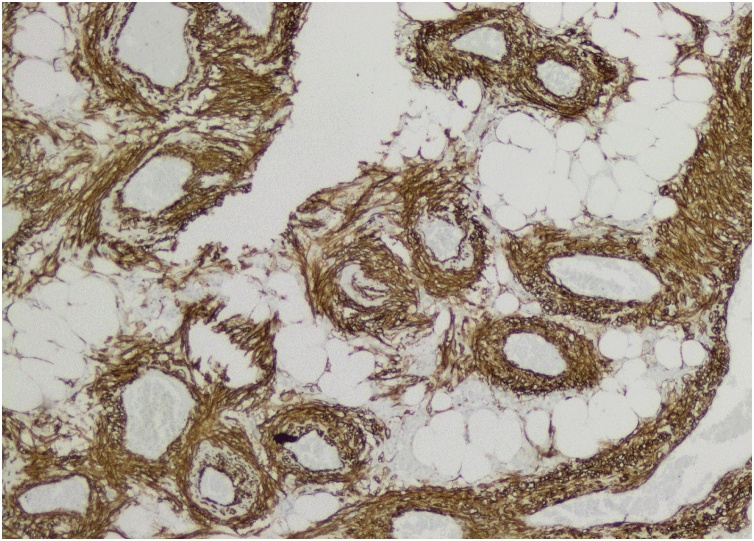
Positive staining of smooth muscle component and of vessel walls with anti-SMA antibodies.

**Fig. 9:**
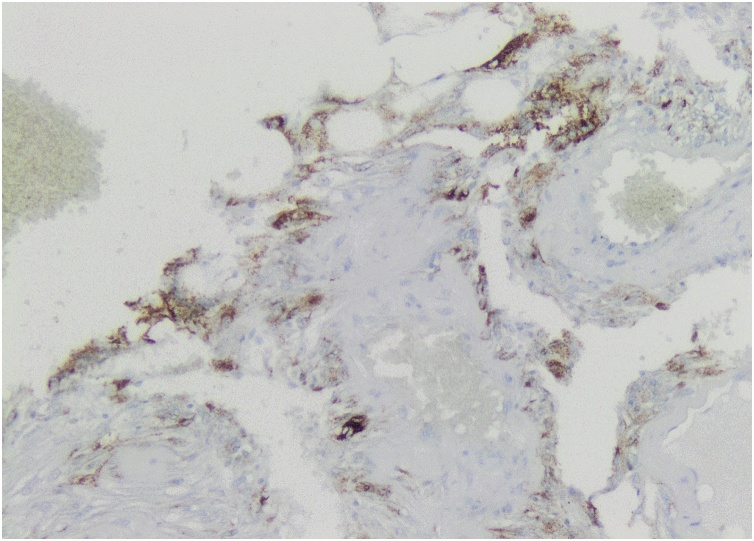
Positive staining of tumor cells with anti-HMB45 antibodies, pathognomonic of AML.

**Fig. 10:**
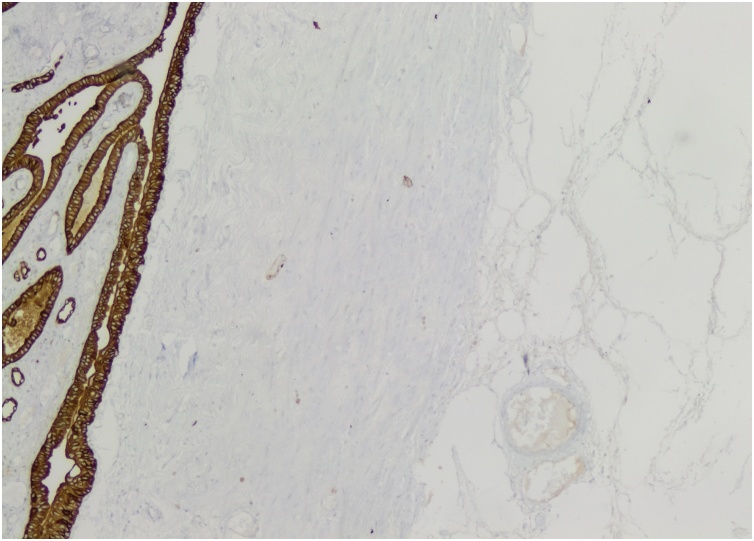
The normal renal tissue stains positive for anti-CK antibodies. This stain marking the epithelial component is negative in the tumor tissue. This marker would have been positive in the tumor in case of renal cell carcinoma or epitheloid AML.

Pathological analysis of the nephrectomy piece revealed a 5 × 4 × 3 cm tumor with intratumoral hemorrhage extending through the hilum by an 8 cm chaplet like structure with diameters varying between 0.8 and 2 cm. Microscopic examination revealed a stroma composed of mixed layers of mature adipocytes and smooth muscle cells and multiple vascular structures with thick walls and hemorrhagic foci. These characteristics were compatible with that of a benign AML, which was confirmed by immunohistochemistry: positive staining with anti-SMA antibodies (Fig. 8) and anti-HMB45 and (Fig. 9), and negative staining with anti-CD34 and anti-CKAE1/AE3 antibodies (Fig. 10).

## 3 Discussion

Renal angiomyolipomas are benign tumors found sporadically in 80% of cases, at an average of 52 years of age, or in associated with tuberous sclerosis complex in the remainder of cases, occurring bilaterally [3].

Uniquely, and opposed to the benign nature of AML, our patient was found with an IVC tumoral invasion. The first case in the literature of a renal AML presenting with a venous thrombus was reported by Kutcher et al. in 1982 and 89 other cases followed, to the best of our knowledge [4].

The majority of cases were female. AML tumors are usually found incidentally on ultrasound, being the primary method of investigation in abdominal pain, but can only visualize the IVC in 60% of cases [5], thus the necessity of a CT scan.

Staging an AML thrombus in the venous system is primordial to assess the therapeutical options. A description of the extent of the thrombus in the IVC lumen is necessary to the staging process, and as by the TNM classification, a T3b tumor is below the diaphragm and a T3c tumor is above the diaphragm [6]. However, Neves, Novick and Hinman have suggested different surgical grades classifications (Table 1), always keeping in mind that the presence of a thrombus in the renal vein or IVC in any type of renal tumor is an indication of complete nephrectomy and thrombectomy [7]. It is recommended to perform a CT scan within 2 weeks of the planned surgery [8].

**Table 1:** Different staging systems for venous thrombis in renal neoplasm. IVC: inferior vena cava RV: renal vein. HV: hepatic vein.

In conclusion, to our knowledge, only 76 cases of AML with IVC invasion were published so far. Malignant transformation of AML should always be kept in mind especially in case of venous invasion. More focus should be put on the ability of AML to invade venous structures despite its benign characteristics. Early imaging and therapeutic planning are necessary for the best outcome. Collaboration of the urologist and the vascular surgeon is sometimes mandatory for optimal surgical treatment.

## Declaration of Competing Interest

No conflict of interest.

## Funding source

No sponsor for this article.

## Ethical approval

This work is exempt from ethical approval in our institution because of its type.

## Consent

Written informed consent was obtained from the patient forpublication of this case report and accompanying images. A copy of the written consent is available for review by the Editor-in-Chief of this journal on request.

## Author contribution

Kheir, El Khoury, El Hachem and Abdessater conceived of the presented idea and were encouraged by Tawil to execute it. El Khoury, Akiel, Abdessater, Kheir and Tawil were on the operating field while performing the surgery and all of them participated to the different steps of the surgery but El Khoury and Tawil were the main surgeons. Kheir, El Khoury, Akiel and Abdessater chose the most important figures from the surgery’s video and pathology. El khoury, Abdessater, El Hachem, Akiel and El Khoury contributed to the final version of the manuscript. Tawil supervised the work. Kheir took the lead in writing the manuscript when he found that this case deserve to be published. All authors provided critical feedback and helped shape the manuscript. The 7 authors designed the model and the computational frame-work and analyzed the results. All persons who meet authorship criteria are listed as authors,and all authors certify that they have participated sufficiently in the work to take public responsibility for the content.

## Registration of research studies

This is not a first-in-man study so it was not registered.

## Guarantor

Raghid El Khoury and Nabil Tawil are the guarantors of this work.

## Provenance and peer review

Not commissioned, externally peer-reviewed.
